# Generation and characterization of an antagonistic monoclonal antibody against an extracellular domain of mouse DP2 (CRTH2/GPR44) receptors for prostaglandin D_2_

**DOI:** 10.1371/journal.pone.0175452

**Published:** 2017-04-10

**Authors:** Nanae Nagata, Hiroko Iwanari, Hidetoshi Kumagai, Osamu Kusano-Arai, Yuichi Ikeda, Kosuke Aritake, Takao Hamakubo, Yoshihiro Urade

**Affiliations:** 1 International Institute for Integrative Sleep Medicine (WPI-IIIS), University of Tsukuba, Tsukuba, Japan; 2 Department of Molecular Behavioral Biology, Osaka Bioscience Institute, Furuedai, Suita, Osaka, Japan; 3 Department of Quantitative Biology and Medicine, Research Center for Advanced Science and Technology, The University of Tokyo, Tokyo, Japan; 4 Department of Advanced Clinical Science and Therapeutics, The University of Tokyo, Tokyo, Japan; 5 Department of Cardiovascular Medicine, Graduate School of Medicine, The University of Tokyo, Tokyo, Japan; Alexion Pharmaceuticals Inc, UNITED STATES

## Abstract

Prostaglandin D_2_ (PGD_2_) is a lipid mediator involved in sleep regulation and inflammation. PGD_2_ interacts with 2 types of G protein-coupled receptors, DP1 and DP2/CRTH2 (chemoattractant receptor homologous molecule expressed on T helper type 2 cells)/GPR44 to show a variety of biological effects. DP1 activation leads to Gs-mediated elevation of the intracellular cAMP level, whereas activation of DP2 decreases this level via the Gi pathway; and it also induces G protein-independent, arrestin-mediated cellular responses. Activation of DP2 by PGD_2_ causes the progression of inflammation via the recruitment of lymphocytes by enhancing the production of Th2-cytokines. Here we developed monoclonal antibodies (MAbs) against the extracellular domain of mouse DP2 by immunization of DP2-null mutant mice with DP2-overexpressing BAF3, murine interleukin-3 dependent pro-B cells, to reduce the generation of antibodies against the host cells by immunization of mice. Moreover, we immunized DP2-KO mice to prevent immunological tolerance to mDP2 protein. After cell ELISA, immunocytochemical, and Western blot analyses, we successfully obtained a novel monoclonal antibody, MAb-1D8, that specifically recognized native mouse DP2, but neither human DP2 nor denatured mouse DP2, by binding to a particular 3D receptor conformation formed by the N-terminus and extracellular loop 1, 2, and 3 of DP2. This antibody inhibited the binding of 0.5 nM [^3^H]PGD_2_ to mouse DP2 (IC_50_ = 46.3 ± 18.6 nM), showed antagonistic activity toward 15(R)-15-methyl PGD_2_-induced inhibition of 300 nM forskolin-activated cAMP production (IC_50_ = 16.9 ± 2.6 nM), and gave positive results for immunohistochemical staining of DP2-expressing CD4+ Th2 lymphocytes that had accumulated in the kidney of unilateral ureteral obstruction model mice. This monoclonal antibody will be very useful for *in vitro* and *in vivo* studies on DP2-mediated diseases.

## Introduction

Prostaglandin D_2_ (PGD_2_) is one of the major cyclooxygenase metabolites and shows its bioactivity via 2 distinct types of G protein-coupled receptors (GPGRs), DP1 and DP2/CRTH2 (the chemoattractant receptor-homologous molecule expressed on Th2 cells)/GPR44. DP1 activation leads to Gs-mediated elevation of the intracellular level of cAMP, whereas activation of DP2 decreases this level via the Gi pathway, and also induces G protein-independent, arrestin-mediated cellular responses [1–3]. In mouse models of allergic asthma or atopic dermatitis, DP2 activation results in eosinophilia and exacerbates the pathology [4–6]. In a previous study, we focused on the physiological function of PGD_2_-DP signaling in a mouse unilateral ureteral obstruction (UUO) model, and found that PGD_2_ contributes to the progression of renal fibrosis via DP2-mediated activation of Th2 lymphocytes [7].

Here, we sought to develop monoclonal antibodies (MAbs) that could compete with PGD_2_ on binding to DP2 receptor. However, it is difficult to develop high-affinity antibodies against the extracellular domain of membrane-integrated DP2 receptors since its 4 extracellular loops are thought to exist in a tightly packed conformation. In this study we used mouse DP2-overexpressing BAF3 cells as an immunogen, immunized DP2-null mutant mice with these cells, and successfully generated an antagonistic monoclonal antibody that recognized the extracellular domain of mouse DP2 and inhibited the binding of PGD_2_ to DP2.

## Materials and methods

### Establishment of MAbs against the extracellular domain of mDP2

#### Construction of plasmids

The cDNA for an HA-tag mDP2 was amplified from reverse-transcribed total RNA extracted from a mouse brain, with amplification done by using sense (5’-tacgctgccaacgtcacactgaagccgctctgt-3’) and antisense (5’-gtcgactcagaccctctgtgggacctctgcactgcc-3’) primers. The amplicon was then subcloned into a pGEM-T vector (Promega, Madison, WI, USA) for sequencing. The cDNAs obtained were cloned between the EcoRV and the SalI sites of the pCXN2/HA vector (kindly provided by Dr. Jun-ichi Miyazaki of Osaka University).

#### Cell culture and transfection for establishment of MAbs

To establish cell lines stably expressing mDP2, we transfected BaF3 and HEK293 cells with an mDP2-containing expression vector by using Lipofectamine (Life Technologies Japan, Tokyo, Japan) according to the manufacturer's instructions. BaF3 cells (Acc. No. RCB0805, RIKEN BRC, Ibaraki, Japan) were cultured in RPMI-1640 medium supplemented with 1 ng/ml mouse IL-3 (R&D Systems, Minneapolis, MN). Following transfection, cells expressing mDP2 were selected with 400 μg/ml of G418 (NACALAI TESQUE, Kyoto, Japan). The generation of viruses, culturing of Sf9 cells, and preparation of budded baculovirus were performed as previously published [4, 8].

#### Cells and plasmids

To establish CHO cells stably expressing mDP2, mDP2 cDNA was subcloned into a pMXs-IRES-Puro vector, which was then used to transfect cells of the retrovirus packaging cell line Plat E [9]. The supernatant from the transfected cells was removed 48–72 h later and applied to CHO cells stably expressing the cAMP response element (CRE)[10]. Transfected cells were selected and maintained in DMEM supplemented with 5% FCS and 1% nonessential amino acids (NACALAI TESQUE) in the presence of hygromycin B (250 μg/ml) and puromycin (10 μg/ml).

#### Animals

Mice were maintained under specific pathogen-free conditions in isolated cages with a 12 h light / 12 h dark photoperiod in a humidity- and temperature-controlled room (55% at 24°C). Water and food were available ad libitum. Mice were anesthetized using isoflurane/O2. All animal protocols in this study were fully conformed to the guidelines outlined in the Guide for the Care and Use of Laboratory Animals of Japan and were approved by the University of Tokyo Animal Care Committee (approval No. RAC130109) and the Animal Research Committee of Osaka Bioscience Institute. The health of mice was monitored one time per week. Every effort was made to minimize the number of animals used and their suffering.

#### Establishment of MAbs

Monoclonal anti-mouse DP2 (mDP2) extracellular domain antibodies were established by modifying the procedures used previously [11, 12]. In brief, the cDNA encoding mDP2 was inserted into a pCXN2/HA transfer vector. DP2-null mice (kindly provided by Dr. Masataka Nakamura, Tokyo Medical and Dental University, Tokyo, Japan) were back-crossed to Balb/c mice for MAb generation in Osaka Bioscience Institute, and the offspring were crossed with Gp64 transgenic mice. To reduce the generation of antibodies against the host cells, and to prevent immunological tolerance to mDP2 protein, these mice (n = 2) were immunized intraperitoneally with mDP2-expressing BaF3, murine interleukin-3 dependent pro-B cells (1x10^7^ cells/mouse) in the presence of budded baculovirus and pertussis toxin. Fusion with NS-1 myeloma cells was carried out by using standard methodology [13]. For the primary screening of hybridomas by cell ELISA and flow cytometry, adsorption with HA-peptide was used to remove the clones that reacted with HA; and 1 clone, D5203-1D8 (MAb-1D8) was obtained.

#### Hybridoma screening

Screening of hybridoma cells by cell ELISA with antigen-expressing COS7 cells was performed as described previously [11]. Antigen-expressing COS7 cells were cultured in poly-D-lysine-coated wells of a 96-well plate (BIOCOAT Poly-D-lysine-coated plate; Falcon 354461) at a density of 1×10^5^ cells/well. After the cells had been incubated with the culture supernatant of hybridoma cells and 20 μg/ml HA-peptide for 1 h, anti-mouse IgG conjugated with horseradish peroxidase was applied; and the enzyme activity was detected by using the substrate tetramethyl benzidine.

The culture supernatant of hybridoma cells was incubated with HEK293 cells stably expressing HA-tagged mDP1 or HA-mDP2 in PBS with 0.1% BSA in the presence of 20 μg/ml HA-peptide on ice for 1 h. After the cells had been washed with 0.1% BSA in PBS, they were incubated with PE-conjugated goat anti-mouse IgG (1:100, Biolegend, San Diego, CA, USA) for 1 h, and then analyzed by flow cytometry with an FACS Calibur flow cytometer (Becton Dickinson, Franklin Lakes, NJ, USA).

For the *in vitro* characterization of monoclonal antibodies against the extracellular domain of mDP2, CHO cells stably expressing FLAG-tagged mDP2 were suspended in PBS with 0.1% BSA and incubated with either 1 μg of anti-FLAG antibody (Sigma, St. Louis, MO, USA) or anti-mDP2 extracellular domain antibodies on ice for 30 min. The cells were then washed with PBS/0.1% BSA and stained by incubation for 30 min with Alexa Fluor 488-conjugated mouse anti-mouse IgG antibody (1:1000, Life Technologies Japan).

#### Immunofluorescence microscopy

HA-mDP1- or HA-mDP2-expressing HEK293 cells were seeded on glass coverslips, fixed with 4% formaldehyde in PBS, and incubated with 2% normal donkey IgG for 30 min at room temperature, and subsequently with the anti-mDP2 extracellular domain antibodies and rat anti-HA antibody (3F10, Roche Diagnostics, Tokyo, Japan) for 24 h at 4°C. The cells were then sequentially incubated with Alexa Fluor 488-conjugated donkey anti-mouse IgG and Alexa Fluor 594-conjugated donkey anti-rat IgG (Life Technologies Japan).

For tissue staining, C57BL/6 mice were purchased from SLC Japan (Japan SLC, Shizuoka, Japan). Unilateral ureteral obstruction (UUO) mice were generated by ligation of the left ureter as described previously [14]. Briefly, at 10 weeks of age, the left ureter was exposed through a lateral incision and ligated with 3–0 silk under isoflurane anesthesia. In sham mice, the left ureter was exposed but not ligated (n = 3 per group). Mice recovered consciousness quickly after the operation. The health of mice was monitored every day in the first week after operation and then one time per week. There were no unexpected deaths and no mice undergoing euthanization with serious infection. Ten days after UUO, the kidneys were harvested from each mouse under deep anesthesia with isoflurane, fixed in 4% paraformaldehyde and embedded in OCT compound (Sakura Finetek Japan, Tokyo, Japan). Cryosections (10-μm thickness) were prepared, incubated with a biotin blocking system solution (Avidin/Biotin Blocking Kit, Vector Laboratories, Burlingame, CA, USA), and then stained by using a M.O.M. Immunodetection Kit (Vector Laboratories), followed by Alexa-Fluor 488-conjugated Streptavidin (Life Technologies Japan) for 1 h at room temperature and then eFluor 615-conjugated rat anti-mouse CD4 (eBioscience, Santa Clara, CA, USA) for 1 h at room temperature. The immuno-stained tissues were observed with an Axiovert 100M microscope connected to a Zeiss laser-scanning microscope 510META (Carl Zeiss, Jena, Germany) and an ECRIPSE Ti (Nikon, Tokyo, Japan).

#### Western blotting

mDP2-expressing HEK293 cells were resuspended in 20 mM HEPES (pH 7.4) with 2 mM EDTA and disrupted with a Dounce homogenizer by 10 up-and-down strokes of a tight-fitting pestle. The homogenates were centrifuged at 1000 x g for 5 min, and the resultant supernatants were further centrifuged at 20,000 x g for 30 min. The pellet was recovered as the membrane fractions and lysed with 1% Triton X-100 in 20 mM Tris/Cl (pH7.4) with 1 mM EDTA containing Complete^™^ protease inhibitor cocktail (Roche Diagnostics).

Samples were incubated at 37°C for 30 min in 0.5 M Tris/Cl (pH8.6) containing 0.5% SDS and 0.1 M 2-mercaptoethanol (2-ME) and then treated at 37°C for 24 h. with N-glycosidase F (PNGase F, Takara Bio, Shiga, Japan) in 100 mM Tris/Cl (pH8.6) containing 1% NP40. Subsequently, the samples were dissolved in 62.5 mM Tris / Cl (pH 6.8) containing 2% (w / v) SDS, 15% (v / v) glycerol, and 5% (v / v) 2-ME, and separated by SDS-PAGE through a 10% SDS-polyacrylamide gel containing 5 M urea, followed by blotting onto a PVDF membrane (Immobilon P; Millipore, Bedford, MA, USA). Samples were not boiled prior to SDS-PAGE, because heating samples results in subsequent aggregation and retention in the stacking gel during electrophoresis [15]. The blots were then incubated with anti-mDP2 or anti-HA antibodies (3F10, Roche Diagnostics). After washing, the blots were incubated with sheep anti-mouse IgG or anti-rat IgG conjugated with horseradish peroxidase (GE healthcare, Waukesha, WI, USA), respectively. Immunoreactive signals were detected by using the ECL Prime Western Blotting Detection System (GE healthcare) with an Image Quant 400 system (GE healthcare).

#### Epitope mapping

Swapped mutants of mDP2 were constructed as follows: The homologous extracellular region of human DP2 (hDP2) was amplified by PCR. The PCR fragments were connected to mDP2 by PCR and subcloned into a pMXs-IRES-Puro vector to establish stably expressing FLAG-tagged mDP2 CHO cells. Then the cells were stained with mDP2-specific antibody MAb-1D8 and rat anti-FLAG antibody (Biolegend) followed by Alexa Fluor 488-conjugated anti-mouse IgG (Life Technologies Japan) and RPE-conjugated anti-rat IgG (Bethyl Laboratories, Montgomery, TX, USA), respectively. Interaction between MAb and swapped mutants was analyzed by flow cytometry as described above.

#### Radioligand binding assay

mDP2-expressing CHO cells at approximately 2×10^5^ cells/well were incubated on ice for 1 h with antibodies (0–3 μM), PGD_2_, or CAY10471 (Cayman Chemical, Ann Arbor, MI, USA) and subsequently for 1 h with 0.5 nM of [^3^H]PGD_2_ in 100 μl of 50 mM Tris-HCl (pH 7.4) containing 10 mM MgCl_2_, 10 mM NaCl, and 0.1% BSA. The cells were then collected on GF/C glass fiber filters (PerkinElmer, Waltham, MA, USA) by vacuum filtration (FILTERMATE 196, PerkinElmer), and the radioactivity captured on the filters was measured by liquid scintillation counting (LSC-6100, Hitachi; Tokyo, Japan).

#### Luciferase reporter assay

CHO cells stably expressing mDP2 and CRE were plated in 96-well plates (30,000 cells/well) and cultured for 24 h. The cells were incubated at 37°C for 1 h with anti-mDP2 antibody or normal mouse IgG and thereafter for 6 h with 300 nM forskolin (NACALAI TESQUE, Kyoto, Japan) and 0.1 nM 15(R)-15-methyl PGD_2_ (Cayman Chemical), a DP2 agonist. Following incubation, firefly luciferase assays were performed by using the Steady-Glo Luciferase Assay System (Promega). Luminescence was detected with a plate reader (ARVO-MX: PerkinElmer) according to the manufacturer’s instructions. EC_50_ values were calculated by using SIGMA PLOT (HULINKS, Tokyo, Japan) [16].

#### Effects of mDP2 MAb on β-arrestin recruitment

HEK293T cells were stably transfected with β-arrestin-1/2-ω chimeras (ω: M15 deletion mutant of β-galactosidase) to establish a reporter cell line (293T/βα-M15). 293T/βα-M15 cells were transiently transfected with mDP2-α chimera (α: a short α peptide fragment of β-galactosidase). After 24 h, the cells were plated in 96-well plates (30,000 cells/well) and cultured for 24 h. Next, the cells were incubated at 37°C for 1 h with anti-mDP2 antibody or normal mouse IgG and then for 90 min with 1 nM DP2 agonist, 15(R)-15-methyl PGD_2_, in OPTI-MEMI/0.1% BSA. Gal-Screen (Life Technologies Japan) substrate was added to the wells, and the plates were further incubated for 90 min at 25°C. Luminescence was measured with a plate reader (ARVO-MX; Perkin Elmer) [16].

### Statistical analyses

All results were expressed as the mean ± SEM. Data involving multiple groups were assessed by ANOVA with Dunnett’s multiple comparison of means test.

## Results

### Characterization of MAbs against the extracellular domain of mDP2

**Fig 1:**
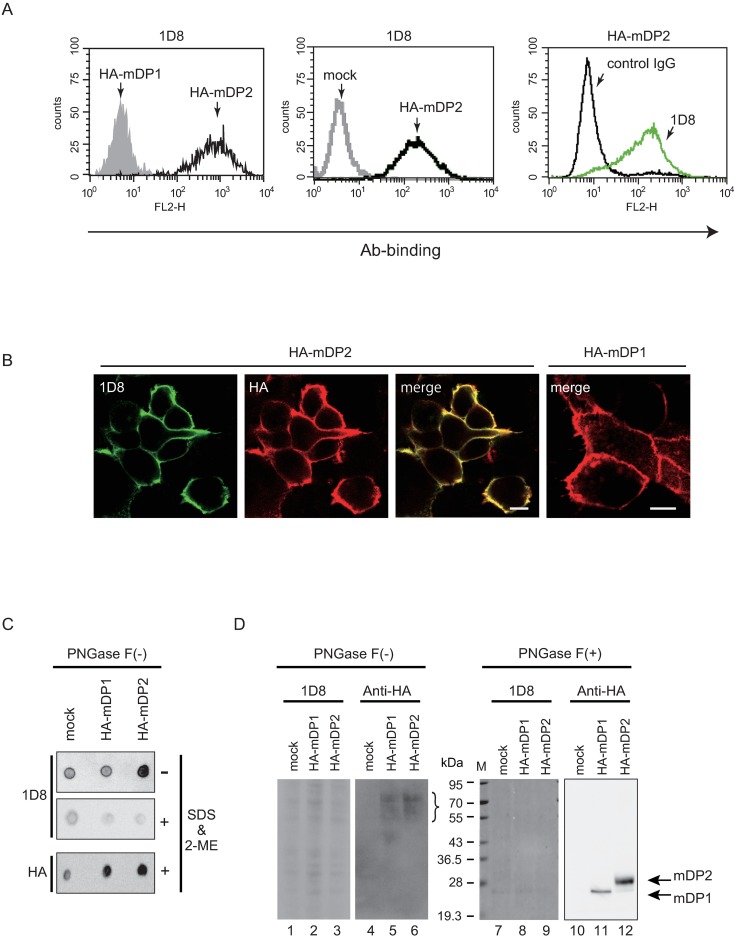
Characterization of anti-mDP2 MAbs. (A) Flow cytometric analysis using MAb-1D8 for binding to HA-mDP1- or HA-mDP2-expressing HEK293 cells. The gray and open zones of the histograms represent HA-mDP1 and HA-mDP2 cells, respectively (left). Comparison of the reactivity of MAb-1D8 between mock cells and HA-mDP2 transfected HEK293 cells. The gray line indicates mock cells and black line indicates the HA-mDP2 expressing cells (middle). Comparison of the reactivity against HA-mDP2-expressing HEK293 cells between control IgG and MAb-1D8. The black line indicates control IgG and green line indicates the reactivity of MAb-1D8 (right). (B) Double immunofluorescence for HA-mDP2 or HA-mDP1 with MAb-1D8 (green) and anti-HA antibody (red). Scale bar, 10 μm. (C) Digitized images of the results of a dot blot assay of the membrane fractions of HA-mDP1- or HA-mDP2-expressing HEK293 cells. (D) Western blot analyses of MAb-1D8. The membrane fractions of HA-mDP1- or HA-mDP2-expressing HEK293 cells were treated with N-glycosidase F or not, separated by SDS-PAGE, and analyzed by Western blotting with each MAb. Lanes 1–3, 7–9: MAb-1D8 reactivity against each sample. Lanes 4–6, 10–12: anti-HA antibody immunoblot reactivity. Positions of molecular size markers are shown on the left.

To develop functional monoclonal antibody associated with antagonistic activity against mDP2, we immunized Gp64-transgenic DP2-null mice by intraperitoneal injection with mDP2-overexpressing BAF3 cells. To assess the specificity of the obtained clones, we used the most closely related GPCR, mDP1 as a reference, since the primary structure of mDP2 exhibits only 25.8% identity to mDP1 (Acc. No. NM009962 for mDP2 and NM008962 for mDP1). Adsorption with HA-peptide was used to remove the clones that reacted with HA. After the primary screening of hybridomas by cell ELISA and flow cytometry, 1 clone, MAb-1D8 (IgG_2a_), was obtained. This clone recognized only mDP2 as judged by flow cytometry using HEK293 cells. MAb-1D8 bound to HA-mDP2 but not to HA-mDP1 or mock-transfected cells (Fig 1A). Control IgG did not bind HA-mDP2-transfected cells. These results indicate that MAb-1D8 was specific for the mDP2 receptor protein and not the HA-tag.

Immunofluorescence analysis revealed that MAb-1D8 clearly stained the plasma membrane of HA-mDP2-expressing HEK293 cells, which staining was identical to the staining pattern obtained with anti-HA antibodies. However, MAb-1D8 did not recognize HA-mDP1-expressing cells (Fig 1B).

Dot blot assays were performed without N-glycosidase F treatment. Without 2% SDS and 5% 2-ME treatment, MAb-1D8 recognized HA-mDP2 but only slightly bound to mock-transfected cells or HA-mDP1 (Fig 1C, upper panel). On the other hand, with 2% SDS and 5% 2-ME treatment, MAb-1D8 did not show any positive immunoreactivity in mock-, HA-mDP1-, and HA-mDP2 (Fig 1C, middle panel), and anti-HA antibody showed strong immunoreactivity to HA-mDP1 and HA-mDP2 (Fig 1C, bottom panel). After tunicamycin treatment, MAb-1D8 recognized HA-mDP2, suggesting that the N-glycosyl chain is not crucial for the antibody recognition (S1 Fig).

By Western blot analysis, immunostaining with MAb-1D8 did not show any positive band in mock-, mDP1-, and mDP2-transfected HEK293 cells before N-glycosidase F treatment (Fig 1D, lanes 1–3), and anti-HA antibody showed broad immunereactive bands at positions of Mr = 50–70 kDa (Fig 1D, lanes 5 and 6, bracket). mDP2 has 3 N-glycosylation sites in its N-terminus domain. The membrane fractions of mock-transfected or HA-tagged mDP1- or mDP2-expressing HEK293 cells were digested with N-glycosidase F, separated by SDS-PAGE, and analyzed by Western blot analysis. After N-glycosidase F treatment, major anti-HA-immunoreactive bands were detected at positions of predicted sizes for HA-mDP1 (Mr = 30 kDa, Fig 1D, lane 11) and HA-mDP2 (Mr = 40 kDa, Fig 1D, lane 12). On the other hand, MAb-1D8 showed no band for HA-mDP1 or for HA-mDP2 (Fig 1D, lanes 7–9), indicating that MAb-1D8 did not recognize denatured mDP2 [17].

### Characterization of MAb-1D8 as a functional antibody

**Fig 2:**
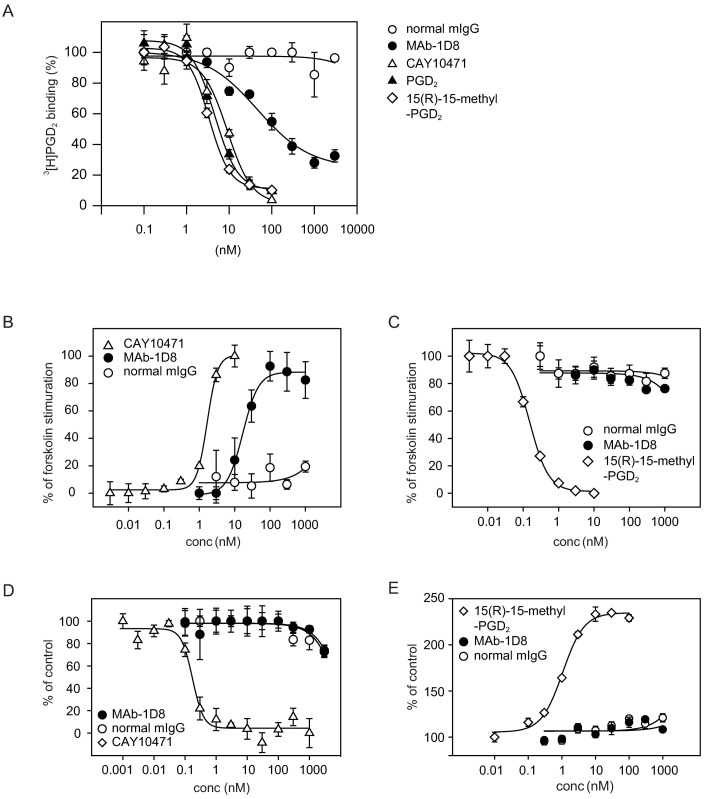
In vitro characterization of MAb-1D8 activity. (A) MAb-1D8-mediated inhibition of 0.5 nM [^3^H]PGD_2_ binding to mDP2 (B and C) Effects of MAb-1D8 on cAMP-production detected by CRE-mediated luciferase activity in mDP2- and CRE-expressing CHO cells. (B) Antagonistic potency of CAY10171, MAb-1D8, and normal mIgG was assessed as the inhibitory activity of 0.1 nM 15(R)-15-methyl PGD_2_ against 300 nM forskolin-activated cAMP production. MAb-1D8 inhibited the decrease in luciferase activity by 15(R)-15-methyl PGD_2_. Forskolin alone was set at 100%. (C) Agonistic potency of 15(R)-15-methyl PGD_2_, MAb-1D8, and normal mIgG was assessed in the presence of 300 nM forskolin. (D) Antagonistic potency of CAY10171, MAb-1D8, and normal mIgG was assessed against 1 nM 15(R)-15-methyl PGD_2_-activated recruitment of β-arrestin in mDP2-expressing 293T/βa-M15 cells. (E) Agonistic potency of 15(R)-15-methyl PGD_2_, MAb-1D8, and normal mIgG on β-arrestin recruitment assay in mDP2-expressing 293T/βa-M15 cells. Representative data (mean ± SEM) from at least 3 independent experiments performed in triplicate are shown.

PGD_2_ is not stable in aqueous solutions and spontaneously dehydrated to form delta12-PGJ_2_ and other PGJ_2_ compounds. Therefore, we used 15(R)-15-methyl PGD_2_ as a stable agonistic analog of PGD_2_ for DP2 [18]. CAY10471 is used as an antagonist of DP2. In an assay to assess the binding of [^3^H]PGD_2_ to mDP2-expressing CHO cells (Fig 2A), PGD_2_, DP2 agonist 15(R)-15-methyl PGD_2_, and DP2 antagonist CAY10471 inhibited the [^3^H]PGD_2_ binding in a concentration-dependent manner, with IC_50_ values of 4.6 ± 0.6 nM, 4.5 ± 1.5 nM and 9.0 ± 3.5 nM, respectively. MAb-1D8 also inhibited the 0.5 nM [^3^H]PGD_2_ binding to mDP2 in a dose-dependent manner, giving an IC_50_ value of 46.3 ± 18.6 nM. On the other hand, normal mIgG did not affected the [^3^H]PGD_2_ binding. In competition binding assays, increasing concentrations of MAb-1D8 did not show a progressive shift of binding curves for either PGD_2_, 15(R)-15-methyl PGD_2_, or CAY10471 (S2A–S2C Fig), suggesting that MAb-1D8 did not change the affinity of the orthosteric ligand for mDP2 at equilibrium, indicative of the neutral cooperativity[19] for this antibody.

### Effects of mDP2 MAb on cAMP-mediated signaling

Next we performed a functional assay using CHO cells stably expressing mDP2 and cAMP response element (CRE)[10]. In the antagonist assay (Fig 2B), DP2 antagonist CAY10471 showed dose-dependent interference (IC_50_ of 1.7 ± 0.1 nM) against the inhibitory activity of DP2 agonist 15(R)-15-methyl PGD_2_ for forskolin-activated cAMP production detected by luciferase activity. MAb-1D8 also showed the interference in a dose-dependent manner, with an IC_50_ of 16.9 ± 2.6 nM; however, normal mIgG had no effect on the luciferase activity. In the agonist assay (Fig 2C), 15(R)-15-methyl PGD_2_ showed dose-dependent inhibition against forskolin-activated cAMP production at IC_50_ of 0.15 ± 0.01 nM. On the other hand, MAb-1D8 and normal mIgG had no effect on cAMP production. These results suggest that MAb-1D8 had antagonistic activity specifically for mDP2.

### Effects of the mDP2 MAb on β-arrestin recruitment

We established a heterologous β-arrestin-GPCR recruitment assay using 293T cells, in which the 1 nM 15(R)-15-methyl PGD_2_-mDP2 interaction was inhibited by CAY10471 in a dose-dependent manner, with an IC_50_ of 0.17 ± 0.03 nM (Fig 2D). However, MAb-1D8 had no effect on this interaction for intracellular β-arrestin recruitment. The agonist activation of mDP2 results in the recruitment of β-arrestin to the receptor at EC_50_ for 15(R)-15-methyl PGD_2_ of 1.1 ± 0.1 nM (Fig 2E). In contrast, MAb-1D8 had no effect on the β-arrestin recruitment at concentrations up to 1 μM.

### Epitope mapping

**Fig 3:**
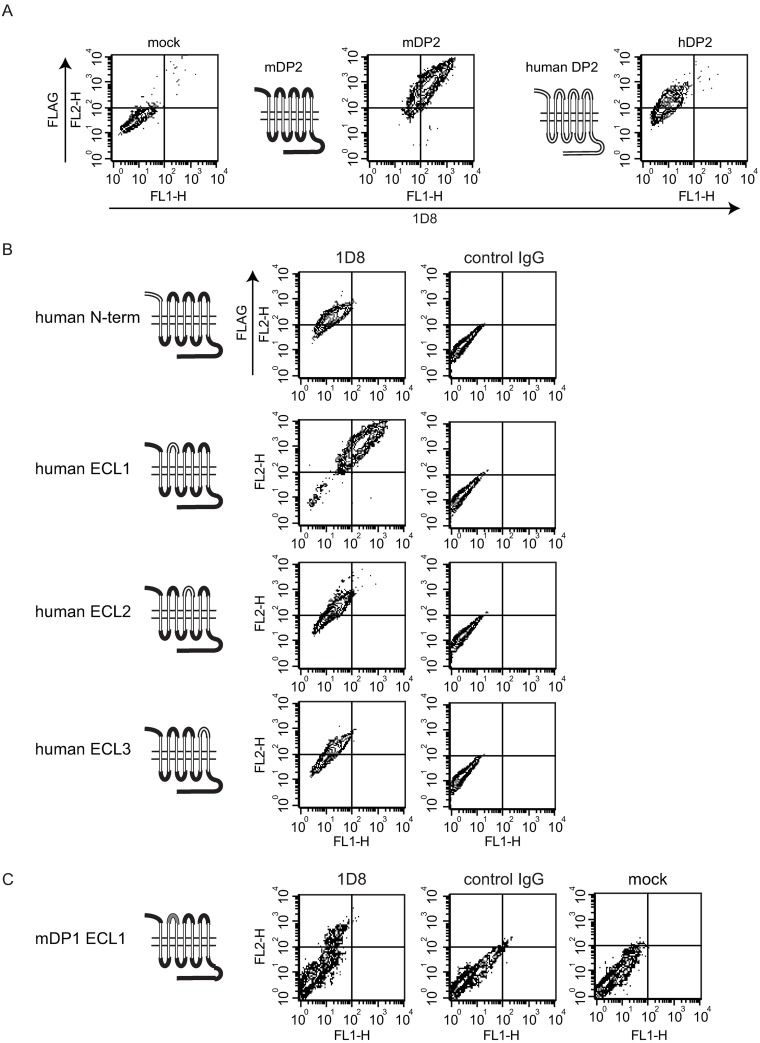
Epitope mapping using swapped DP2 mutants. (A) Flow cytometric analysis using MAb-1D8 and mock transfected (left), FLAG-mDP2- (middle) or FLAG-hDP2- (right) expressing CHO cells. (B) An extracellular domain of mDP2 was swapped with the homologous region of hDP2 by PCR. Flow cytometric analysis was performed using MAb-1D8 and FLAG-tagged wild-type or swapped mDP2 mutant-expressing CHO cells. (C) ECL1 of mDP2 was swapped with the homologous region of mDP1.

As the primary structure of mDP2 exhibits 80.2% identity to hDP2 (75 amino acid substitutions out of a total of 380 amino acid residues), we evaluated whether MAb-1D8 recognized hDP2 stably expressed in CHO cells. Flow cytometry analyses revealed that MAb-1D8 did not recognize hDP2 (Fig 3A). This was in agreement with the result that MAb-1D8 had no effects on hDP2-mediated actions (S3 Fig). To determine the epitope of MAb-1D8, we generated loop-swapped mutants of mDP2 and stably transfected CHO cells with them, in which 1 of the 3 extracellular loops (ECLs) or N-terminus was changed to the corresponding region of hDP2 (Fig 3B). The identity of ECLs for mDP2 (Acc. No. NM009962) to hDP2 (NM004778) was as follows: N-terminus, 69.7%; ECL1, 94.1%; ECL2, 75.0%; and ECl3, 60.9% (S4 Fig). Replacement of the ECL1 of mDP2 with that of hDP2 did not affect the binding of MAb-1D8 to the chimeric DP2, whereas replacement of N-terminus, ECL2 or ECL3 of mDP2 with that of hDP2 resulted in loss of the binding of MAb-1D8 to DP2, indicating that the epitope of MAb-1D8 included N-term, ECL2, and ECL3. Although the identity of ECL1 between mDP2 and hDP2 is 94.1% (1 amino acid substitution out of a total of 17 amino acid residues), the possibility that the epitope recognized by MAb-1D8 included ECL1 still remained. So we generated a mutant in which the ECL1 of mDP2 was changed to the corresponding region of mDP1. The identity of ECL1 between mDP2 and mDP1 (NM008962) was 5.6% (S4 Fig). Replacement of ECL1 of mDP2 with that of mDP1 negated the binding of MAb-1D8 to DP2, indicating that the epitope of MAb-1D8 included ECL1 (Fig 3C).

**Fig 4:**
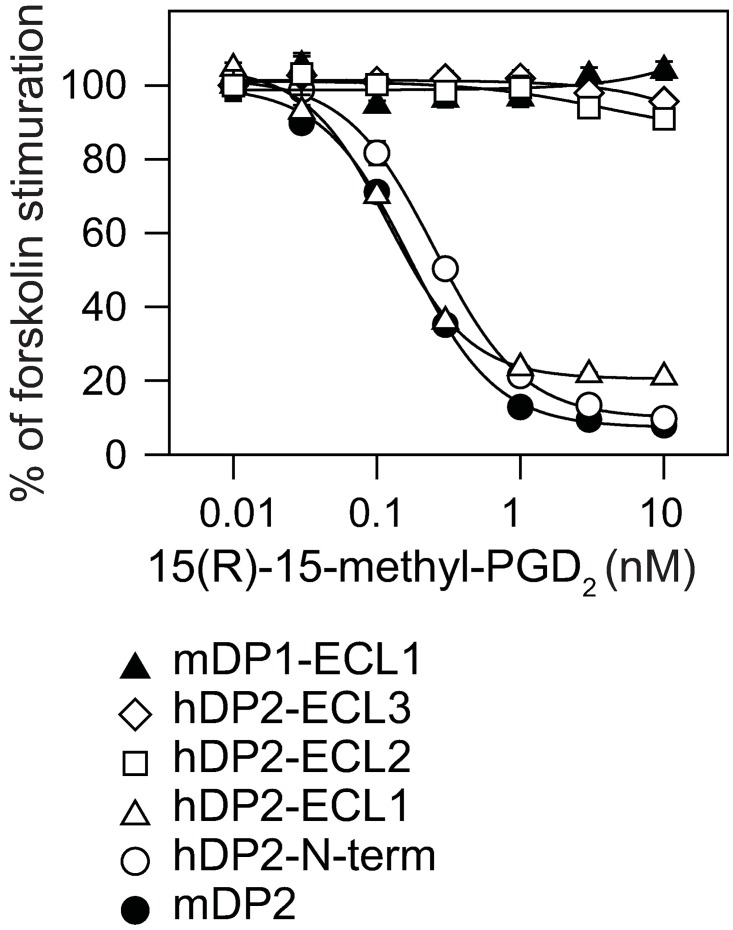
Agonistic potency of 15(R)-15-methyl PGD_2_ on the N-terminus or ECL-swapped DP2-expressing cells. The luciferase activity was assessed in the presence of 300 nM forskolin.

When we examined the functional activity of theses chimeric DP2-expressing cells. 15(R)-15-methyl PGD_2_ dose-dependently inhibited the forskolin-activated cAMP production in N-terminus- or ECL1-swapped mutants (Fig 4). On the other hand, replacement of ECL1 with that of mDP1, or replacement of ECL2 or ECL3 with that of hDP2 diminished the inhibitory effect of 15(R)-15-methyl PGD_2_ on the cAMP production, suggesting that these chimeric receptors lost the functional conformation of DP2.

Taken together, these results reveal that MAb-1D8 recognized the 3D structure composed of N-terminus and 3 ECLs of mDP2, which structure was different between mDP2 and hDP2.

### DP2 immunohistochemistry on UUO kidney

**Fig 5:**
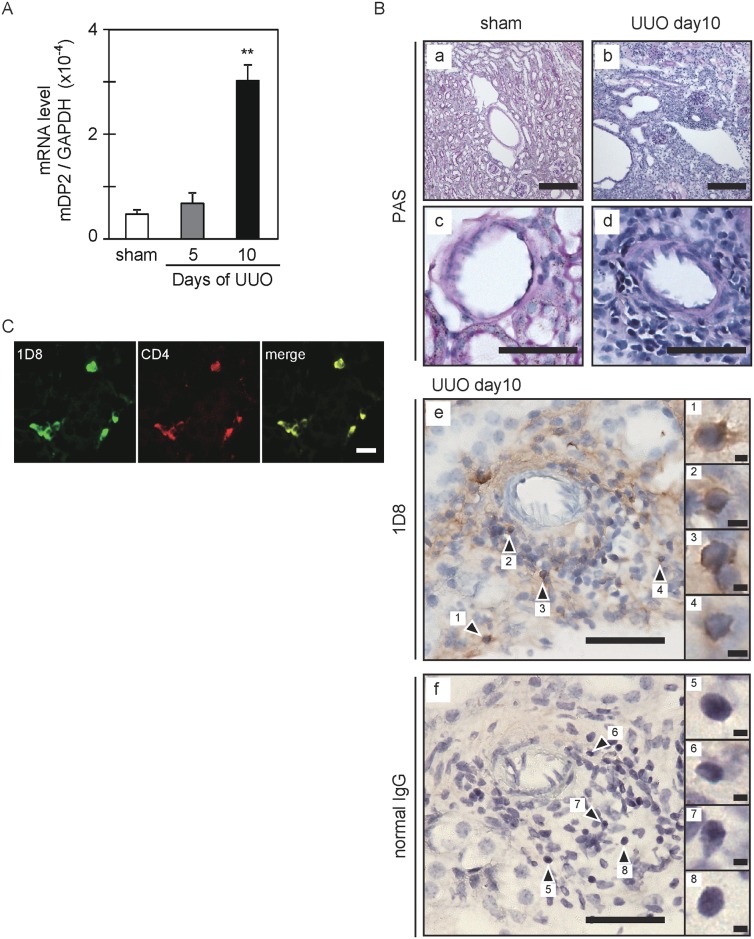
Immunostaining for mDP2 in the UUO kidney at 10 days after obstruction. (A) mDP2 mRNA expression. The copy numbers / 40 ng total RNA were measured by qPCR and used to calculate the ratios. **P < 0.01 (one-way ANOVA). Each value represents the mean ± S.E. (B) Periodic acid–Schiff (PAS) staining and immunostaining for mDP2 with MAb-1D8. Scale bar: 200 μm (a and b), 50 μm (c-f), 2 μm (inset 1–8). (C) Double immunofluorescence for mDP2 with MAb-1D8 (green) and Th2 cells marker CD4 (red). Scale bar: 20 μm.

In a previous study, we demonstrated that PGD_2_ is locally produced after UUO and activates Th2 lymphocytes via DP2 expressed on CD4-positive T cells [7]. In the UUO kidney, the expression of DP2 mRNA was significantly elevated after 10 days (Fig 5A); and many lymphocytes had infiltrated to the interstitium near the blood vessels, as shown by periodic acid–Schiff (PAS) staining (Fig 5Bb and 5Bd). In contrast, the infiltration was scarce in the sham kidney (Fig 5Ba and 5Bc). MAb-1D8 showed positive immunoreactivity (arrowheads) toward infiltrating lymphocytes in the UUO kidney (Fig 5Be). No positive signals were observed in the UUO kidney of DP2-KO mice (S5 Fig). Normal mice IgG showed no positive immunoreactivity in the UUO kidney (Fig 5Bf). Immunofluorescence analyses demonstrated that MAb-1D8-positive fluorescence overlapped with that of CD4, a Th marker, in the UUO kidney (Fig 5C). These results indicate that MAb-1D8 is useful for immunohistological analysis of mDP2-expressing CD4+ cells that migrate into sites of inflammation within a tissue.

## Discussion

In this study, we successfully generated a highly specific functional antibody against mDP2, MAb-1D8, which blocked the binding of PGD_2_ to mDP2 and showed antagonistic activity toward mDP2. DP2 is expressed in immune cells such as Th2 cells, basophils, and eosinophils [20]. Previous reports showed that activation of DP2 by PGD_2_ results in the recruitment of Th2 cells and other leukocytes [2] by driving the production of Th2-cytokines [21]. DP2 has drawn much attention as a therapeutic target for the treatment of inflammatory conditions such as asthma [22–24], allergic rhinitis [25, 26], and atopic dermatitis [27, 28]. In mouse models of allergic asthma or atopic dermatitis, DP2 activation results in eosinophilia and exacerbates the pathology [4–6]. Antagonism of PGD_2_ binding to DP2 may be a suitable strategy to reduce symptoms of Th2-type inflammatory diseases [29]. A functional MAb provides an alternative approach to identifying drugs designed for such targets. MAb-1D8 will be a useful pharmacological tool for elucidating the biological effects of DP2 in various mouse model systems.

Purified full-length membrane protein with the correct 3D structure is regarded as the ideal immunogen for the production of MAb against a membrane protein. However, immunization with cells having a high level of antigen expression has some technical limitations such as low expression level or form aggregates [30–32]. We previously immunized BALB/c mice intraperitoneally with antigen-expressing BaF3 cells and then challenged them once a week up to 20 times with BaF3 cells followed by an interval of 2 weeks to generate monoclonal antibody. However, the efficiency of hybridoma formation specific for the antigen was very low: 0, 1, and 2 FACS-positive clones/1144, 1056, and 2728 hybridomas with 7, 18, and 20 times immunizations, respectively [11]. On the other hand, as shown in the present study, when we immunized gp64-transgenic mice crossed with DP2-null mice by intraperitoneal injections with mDP2-expressing BaF3 cells in the presence of budded baculovirus and pertussis toxin or pertussis toxin alone as adjuvant, the efficiency of hybridoma formation was markedly increased to 12 FACS-positive clones/960 hybridoma with 2 or 3 immunizations. Finally we succeeded in developing a single clone, MAb-1D8, that demonstrated specific binding to and antagonistic activity toward mDP2 (Fig 1A and 1B). MAb-1D8 showed no positive immunoreactivity in the UUO kidney of DP2-KO mice (S5 Fig), suggesting that MAb-1D8 recognized the DP2-expressing CD4+ lymphocyte accumulated in the kidney of UUO model mice (Fig 5).

MAb-1D8 partially inhibited the binding of 0.5 nM [^3^H]PGD_2_ to 32% at concentration up to 3 μM (IC_50_ = 46.3 nM; Fig 2A). In competition binding assays, increasing the concentration of MAb-1D8 did not cause a shift in the binding curve for PGD_2_, 15(R)-15-methyl PGD_2,_ or CAY10471 (S2A–S2C Fig). These results suggest that MAb-1D8 bound to the similar position to which PGD_2_ binds.

Although MAb-1D8 showed no agonistic effect in the cAMP-mediated luciferase reporter (Fig 2C) and β-arrestin recruitment (Fig 2E) assays at concentrations up to 1 μM, it did have an antagonistic effect in the cAMP-mediated luciferase reporter assay (Fig 2B). However, MAb-1D8 had no such effect in the β-arrestin recruitment assay at concentrations up to 1 μM (Fig 2D). The concentration of 15(R)-15-methyl PGD_2_ used in the antagonistic luciferase reporter assay and antagonistic β-arrestin recruitment assay was 0.1 nM and 1 nM, respectively. MAb-1D8 could not completely inhibit the binding of 0.5 nM [^3^H]PGD_2_ at concentration up to 3 μM (Fig 2A), suggesting that the antagonistic activity of MAb-1D8 was not enough to inhibit the β-arrestin recruitment induced by 1 nM 15(R)-15-methyl PGD_2_. This difference in receptor activation by 15(R)-15-methyl PGD_2_ between cAMP signaling (EC_50_ = 0.15 ± 0.01 nM) and β-arrestin recruitment (EC_50_ = 1.1 ± 0.1 nM) is thought to be due to biased agonism [3, 33]. These results suggest that MAb-1D8 could not stabilize the active conformation of receptor that can signal down both pathways.

MAb-1D8 was shown to be conformationally sensitive, as determined by its lack of binding to receptor protein in Western analysis after SDS-PAGE, in which case the receptor protein would be denatured and only linear epitopes exposed (Fig 1D). MAb-1D8 distinguished between human and mouse DP2. Epitope analyses (Fig 3) showed that MAb-1D8 may have recognized a particular 3D conformation formed by the N-terminus and ECL1, 2, and 3 of DP2.

It is reported that ECL2 has an important role in binding of orthosteric and allosteric ligands to GPCRs [34] and that ECL2 is a potent target for activating allosteric antibodies in various Family A receptors [35]. In the calcium-sensing receptor of Family C, the ECL2 is involved in the effects of allosteric modulators [36]. Therefore, immobilization of ECLs by MAb-1D8 would be expected to affect the receptor activities.

According a report appearing in 2012 [37], 28 MAbs, targeting CD20, tumor necrosis factor (TNF), epidermal growth factor receptor (EGFR), vascular endothelial growth factor, and various unique antigens, have been marketed in either Europe or the USA as therapeutic MAbs. Although about 350 mAbs are currently under clinical evaluation [37], the development of a MAb targeting DP2 has not been reported [32]. MAb-1D8 is thus the first functional antibody against PG receptors to be reported. Co-crystallization studies using the Fab of MAb-1D8 might shed light on the crystal structure of mDP2.

In conclusion, we generated a unique antagonistic monoclonal antibody, MAb-1D8, which bound to the extracellular surface of mDP2 and was shown to be suitable for immunohistochemical staining, flow cytometric detection of mDP2 but not for Western Blotting using SDS gels. Therefore, MAb-1D8 should become a useful tool for *in vitro* and *in vivo s*tudies on DP2-mediated diseases.

## Supporting information

S1 Fig Dot blot assay after tunicamycin treatment. For tunicamycin treatment, cells were treated with culture medium containing 5 μg/ml tunicamycin (SIGMA) for 24 h.
(TIF)

S2 Fig Competition binding assays. Effect of MAb-1D8 on the competition between agonist PGD_2_ (A), 15(R)-15-methyl PGD_2_ (B), or antagonist CAY10471 (C) and [^3^H]PGD_2_ for binding of the latter to mDP2. The binding of [^3^H]PGD_2_ in the absence of competitor was set at 100%.
(TIF)

S3 Fig MAb-1D8 had no effects on hDP2-mediated actions. (A) Antagonistic potency of CAY10171, MAb-1D8, and normal mIgG was assessed as the inhibitory activity of 0.3 nM 15(R)-15-methyl PGD_2_ against 300 nM forskolin-activated cAMP production. (B) Agonistic potency of 15(R)-15-methyl PGD_2_, MAb-1D8, and normal mIgG was assessed in the presence of 300 nM forskolin.
(TIF)

S4 Fig Amino acid sequence alignments of extracellular loops of mouse and human DP2. Gaps in the sequences to facilitate alignment are indicated by dashes. Conserved residues (*) are indicated below the sequences.
(TIF)

S5 Fig Immunostaining for mDP2 in the UUO kidney of DP2-KO mice. Scale bar: 20 μm, 2 μm (inset).
(TIF)
