# Supplementary figures and images for: Generation and characterization of an antagonistic monoclonal antibody against an extracellular domain of mouse DP2 (CRTH2/GPR44) receptors for prostaglandin D2
Source: PLoS One. 2017 Apr 10;12(4):e0175452. doi: 10.1371/journal.pone.0175452 (PMC5386288; doi:10.1371/journal.pone.0175452)

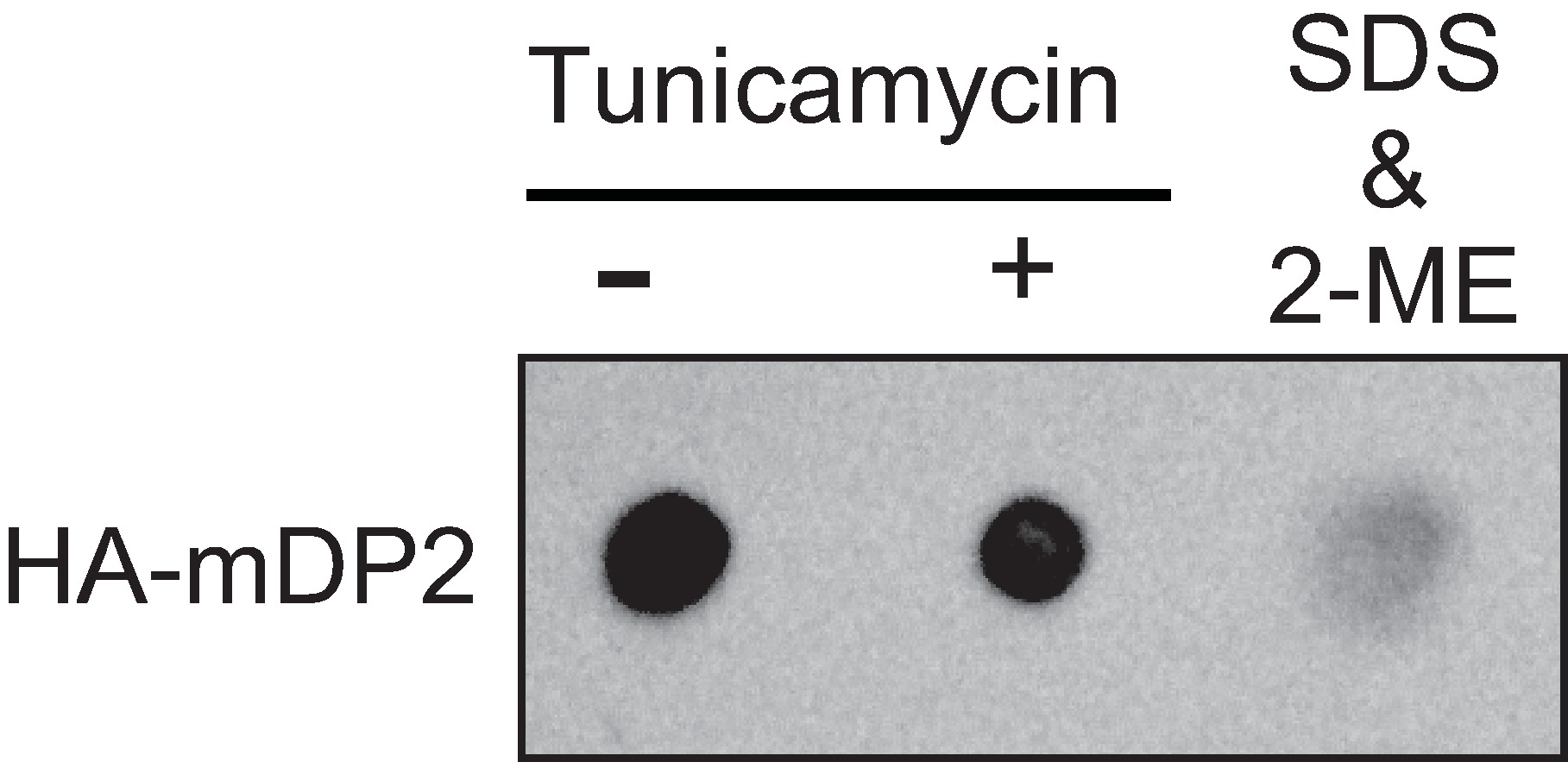

Supplement: S1 Fig — For tunicamycin treatment, cells were treated with culture medium containing 5 μg/ml tunicamycin (SIGMA) for 24 h. (TIF) [file pone.0175452.s001.tif]

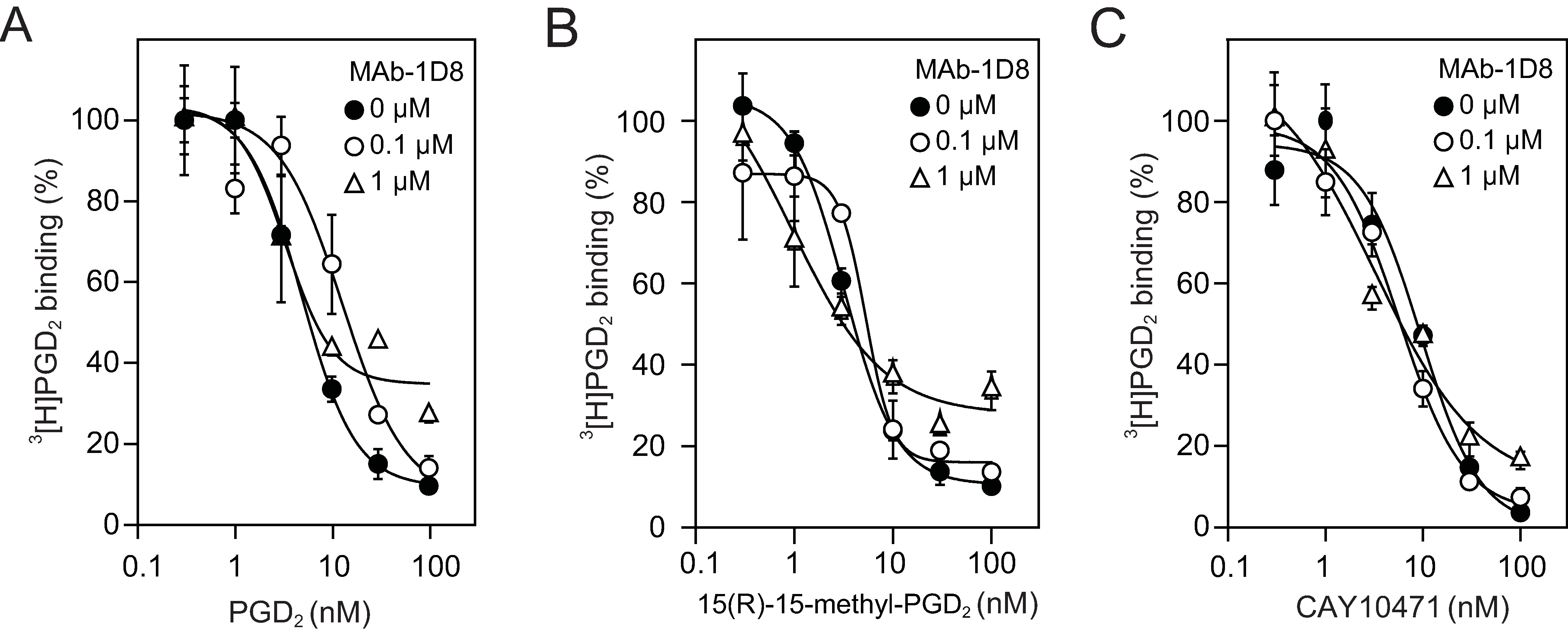

Supplement: S2 Fig — Effect of MAb-1D8 on the competition between agonist PGD2 (A), 15(R)-15-methyl PGD2 (B), or antagonist CAY10471 (C) and [3H]PGD2 for binding of the latter to mDP2. The binding of [3H]PGD2 in the absence of competitor was set at 100%. (TIF) [file pone.0175452.s002.tif]

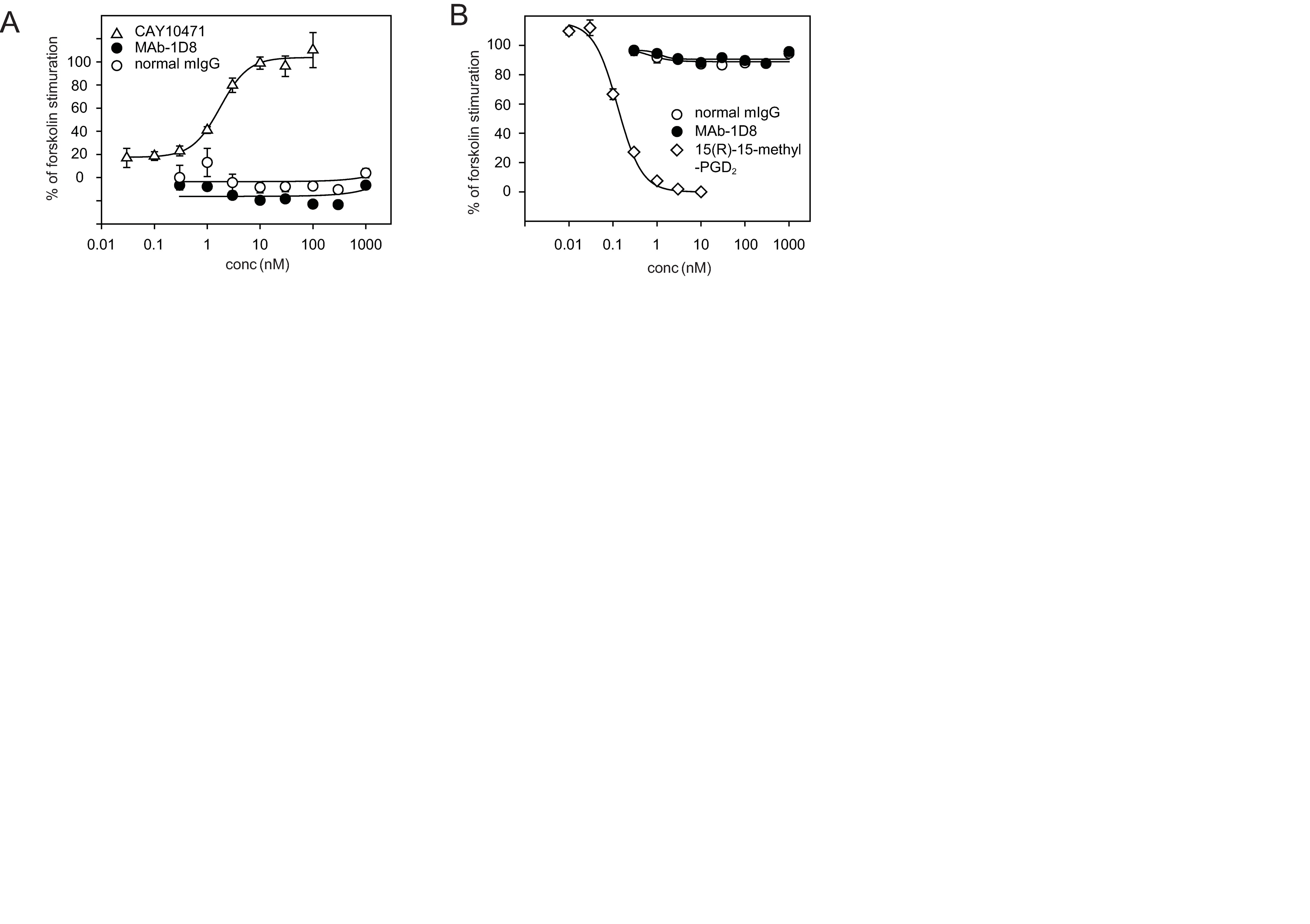

Supplement: S3 Fig — (A) Antagonistic potency of CAY10171, MAb-1D8, and normal mIgG was assessed as the inhibitory activity of 0.3 nM 15(R)-15-methyl PGD2 against 300 nM forskolin-activated cAMP production. (B) Agonistic potency of 15(R)-15-methyl PGD2, MAb-1D8, and normal mIgG was assessed in the presence of 300 nM forskolin. (TIF) [file pone.0175452.s003.tif]

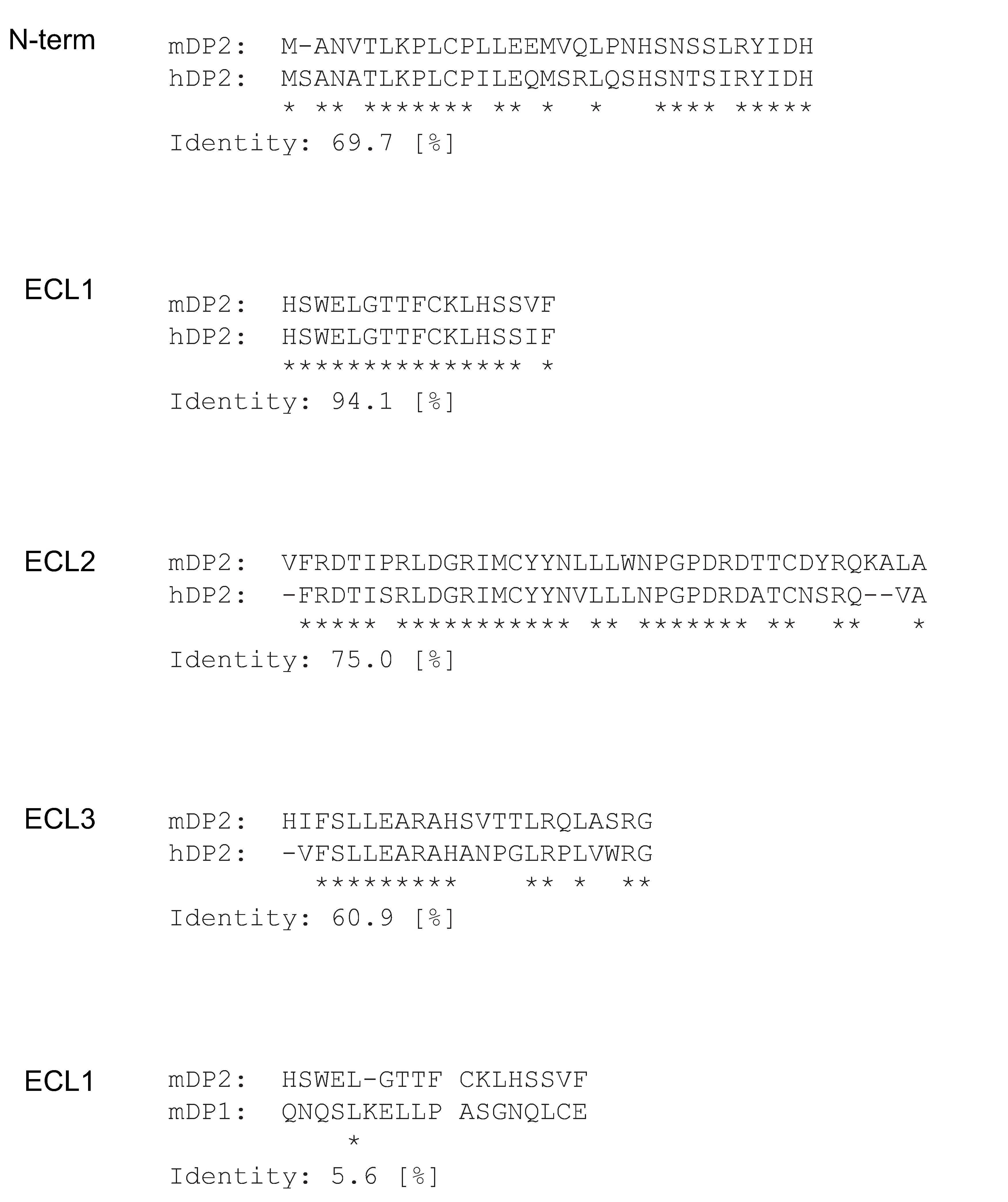

Supplement: S4 Fig — Gaps in the sequences to facilitate alignment are indicated by dashes. Conserved residues (*) are indicated below the sequences. (TIF) [file pone.0175452.s004.tif]

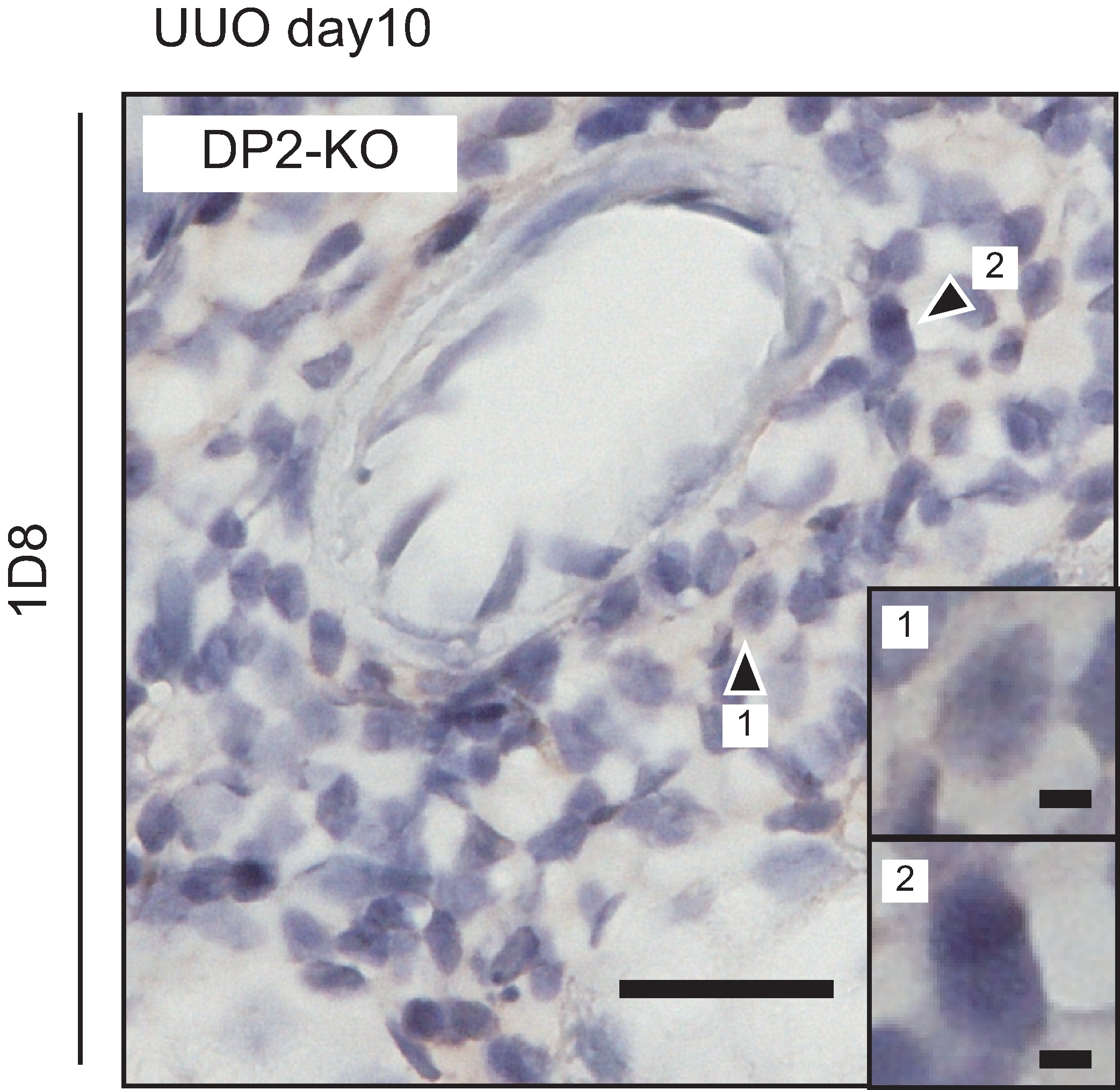

Supplement: S5 Fig — Scale bar: 20 μm, 2 μm (inset). (TIF) [file pone.0175452.s005.tif]
